# Supplementary material for: Topological magnon amplification
Source: Nat Commun. 2019 Sep 2;10:3937. doi: 10.1038/s41467-019-11914-2 (PMC6718654; doi:10.1038/s41467-019-11914-2)
Supplement: Supplementary file 1 — Supplementary Information [file 41467_2019_11914_MOESM1_ESM.pdf]

Supplementary Material: Topological magnon amplification

Malz *et al.*

SUPPLEMENTARY NOTE 1: EFFECTIVE SPIN HAMILTONIAN FROM FERMI-HUBBARD MODEL

In order to support our qualitative analysis above, we derive the polarization tensor in a kagome TMI from a Fermi-Hubbard model at half filling, with an on-site Coulomb repulsion U much larger than the hopping t, $t/U \ll 1$. As is well known, the low-energy physics can be described by perturbing around the Mott insulator state [1]. A contribution to the polarization arises to third order in the hopping [2] (hopping around a triangle), which is derived in detail below.

Following Zhu *et al.* [3], we consider a one-band Hubbard model with SOC

$$H_{\text{Hubbard}} = -\sum_{\langle ij\rangle}\left[\mathbf{c}_i^\dagger\left(\tau_{ij}+\mathbf{d}_{ij}\cdot\boldsymbol{\sigma}\right)\mathbf{c}_j+\text{H.c.}\right]+U\sum_j n_{j\uparrow}n_{j\downarrow}, \tag{1}$$

with $\mathbf{c}_i = (c_{j\uparrow}, c_{j\downarrow})^T$, and SOC vector $\mathbf{d}_{ij} = i\mathbf{n}_{ij}t_{ij}\sin(\theta_{ij})$. The parameterization of $\mathbf{d}$ in terms of a unit vector $\mathbf{n}_{ij}$ and an angle θ_{ij} will become useful later.

Zhu *et al.* [3] derive the effective low-energy spin Hamiltonian to second order in the hopping, which is

$$\tilde{H}_{\text{eff}} = \sum_{\langle ij\rangle}\frac{4t_{ij}^2}{U}\mathbf{S}_i^\dagger\mathcal{J}_{ij}\mathbf{S}_j, \tag{2}$$

where $S_j^\nu = \frac{1}{2}\mathbf{c}_j^\dagger\cdot\sigma^\nu\cdot\mathbf{c}_j$, and $\mathcal{J}_{ij}$ is the exchange tensor pertaining to bond $\langle ij\rangle$ and can be written

$$\mathcal{J}_{ij}\mathbf{S}_j = \cos(2\theta_{ij})\mathbf{S}_j + \sin(2\theta_{ij})(\mathbf{S}_j\times\mathbf{n}_{ij}) + 2\sin^2(\theta_{ij})\mathbf{n}_{ij}(\mathbf{n}_{ij}\cdot\mathbf{S}_j). \tag{3}$$

The three terms give rise to the isotropic Heisenberg interaction, to asymmetric, and to symmetric exchange anisotropy, respectively.

SUPPLEMENTARY NOTE 2: POLARIZATION TENSOR

The direction in which the polarization may point is constrained in the same way as the DM vector associated to each bond [*cf.* Eq. (4) in the main text]. The reflection symmetry around the plane orthogonal to each bond constrains the vector to lie in this symmetry plane. In addition, the lattice is three-fold rotation symmetric, as well as inversion symmetric around lattice sites, such that the direction of one vector determines that of all others. The precise direction and magnitude of the vector may be obtained, for example, from perturbation theory in the Fermi-Hubbard model at half filling, as we demonstrate below. The results in the main text in principle only require that the anisotropic part of the polarization tensor is nonzero, which is allowed whenever bonds are not centres of inversion.

Microscopically, the anisotropy is due to spin-orbit coupling (SOC). We follow Zhu *et al.* [3], who derive the electric polarization as a third-order hopping process, which is the lowest-order relevant contribution. It is given through [3]

$$\mathbf{P}_{ij} = \mathbf{p}_{0,ij}\left[\mathbf{S}_i\cdot\mathcal{J}_{ij}\mathbf{S}_j\cos\theta_{ijk} + \mathbf{n}_{ijk}\cdot\mathbf{S}_i\times\mathcal{J}_{ij}\mathbf{S}_j\sin\theta_{ijk}\right] \tag{4}$$

where k is the third site in the loop and with

$$\mathbf{p}_{0,ij} \equiv 8ea\frac{t_{ij}t_{jk}t_{ki}}{U^3}(\mathbf{e}_{jk}-\mathbf{e}_{ki}) = 8ea\frac{t_{ij}t_{jk}t_{ki}}{U^3}(2\boldsymbol{\rho}_k-\boldsymbol{\rho}_i-\boldsymbol{\rho}_j). \tag{5}$$

The vector $\mathbf{p}_{0,ij}$ points into the triangle, orthogonal to the bond $\langle ij\rangle$ and in the plane of the triangle. Importantly, this means that, when following bonds along a straight line, their polarization changes sign from bond to bond.

The angle θ parametrizes the relative strength of the SOC. To first order in θ, the only scalar quantity one can construct with one vector are of the form $\mathbf{n}\cdot(\mathbf{S}\times\mathbf{S})$, which does not have the form we are interested in. Hence we expand to second order

$$\mathbf{P}_{ij} = \mathbf{P}_{ij}^{(0)} + \mathbf{P}_{ij}^{(1)} + \mathbf{P}_{ij}^{(2)} + \mathcal{O}(\theta^3), \tag{6}$$

with

$$\mathbf{P}_{ij}^{(0)} = \mathbf{p}_{0,ij}\mathbf{S}_i \cdot \mathbf{S}_j, \tag{7a}$$

$$\mathbf{P}_{ij}^{(1)} = \mathbf{p}_{0,ij}\left(2\theta_{ij}\mathbf{n}_{ij} + \theta_{ijk}\mathbf{n}_{ijk}\right)\cdot(\mathbf{S}_i \times \mathbf{S}_j), \tag{7b}$$

$$\mathbf{P}_{ij}^{(2)} = \mathbf{p}_{0,ij}\left\{-(\mathbf{S}_i\cdot\mathbf{S}_j)[2\theta_{ij}^2 + 2\theta_{ij}\theta_{ijk}(\mathbf{n}_{ijk}\cdot\mathbf{n}_{ij}) + \tfrac{1}{2}\theta_{ijk}^2] + 2\theta_{ij}^2(\mathbf{S}_i\cdot\mathbf{n}_{ij})(\mathbf{S}_j\cdot\mathbf{n}_{ij}) + 2\theta_{ij}\theta_{ijk}(\mathbf{S}_i\cdot\mathbf{n}_{ij})(\mathbf{S}_j\cdot\mathbf{n}_{ijk})\right\}. \tag{7c}$$

Physically, in the original Hubbard Hamiltonian, only the SOC term can generate spin flips, which is why we need to go to second order in the SOC to obtain anomalous pairing terms that generate two magnons from one photon. In the spin wave picture, terms such as $(\mathbf{S}_i\cdot\mathbf{n}_{ij})(\mathbf{S}_j\cdot\mathbf{n}_{ij})$ and $(\mathbf{S}_i\cdot\mathbf{n}_{ij})(\mathbf{S}_j\cdot\mathbf{n}_{ijk})$ can lead to anomalous terms, which can lead to instabilities and thus amplification. In order to make progress, we need to apply the general model Eq. (2) to our particular problem. Note that while Eq. (2) predicts a positive J, experiments show that J is in fact negative. This is a result of other contributions, such as exchange. Thus, the measured J cannot be used to determine the angle θ_{ij} in Eq. (2). Instead, the angle needs to be fitted independently, or determined from a measurement of the spin-orbit effect. Comparing Eq. (2) to Eq. (4) in the main text, we can identify

$$\frac{4t_{ij}^2}{U}\cos(2\theta_{ij}) = J_{\mathrm{SE}}, \qquad \frac{4t_{ij}^2}{U}\sin(2\theta_{ij})\mathbf{n}_{ij} = \mathbf{D}_{ij}, \tag{8}$$

where $\mathbf{D}_{ij}$ is the vector in Eq. (4) in the main text, $\tan(2\theta_{ij}) = -|\mathbf{D}_{ij}|/J_{\mathrm{SE},ij}$ quantifies the strength of the DM interaction relative to the Heisenberg coupling from superexchange J_{SE}, and we have used the subscript SE to denote the superexchange contribution. In principle, all these quantities can differ from site to site, but here we study a translation-invariant Hamiltonian, which simplifies the description considerably.

By lattice symmetry, $\mathbf{D}$ has to lie in the plane orthogonal to the bonds (since that is a symmetry plane). In the pyrochlore lattice, each bond is part of two triangles. The net DM interaction is the sum of the contribution from each triangle. If we consider the corner-sharing cube that surrounds the tetrahedron, the DM vector lies in the plane of the cube face that also encompasses the bond, as derived for instance in Ref. 4. If we choose the upright triangles in Fig. 1 in the main text to be part of tetrahedra pointing into the plane (and thus the upside-down triangles are part of tetrahedra pointing out of the plane), and consider the bond lying along x in an upright triangle, we have $\mathbf{n}_{12} = -(\sqrt{2/3})\hat{\mathbf{z}} - 1/\sqrt{3}\hat{\mathbf{y}}$ ($\hat{\mathbf{z}}$ points out of the plane, i.e., our coordinate system is right handed with Fig. 1 in the main text being the xy-plane, with x being horizontal and y vertical). The DM vectors for the other bonds in the triangle can be obtained through rotation by $2\pi/3$ around z. The DM vectors in upside-down triangle then follow from reversing the vectors in the upright triangle ($\mathbf{v}\to-\mathbf{v}$). This argument assumes ordered bonds (here counterclockwise in all triangles).

This determines $\mathbf{n}_{ij}$ and $\theta_{ij}\equiv\theta = (1/2)\tan^{-1}(D/J_{\mathrm{SE}})$. The spin-orbit contribution is assumed to be weak, such that θ is small. In analogy to a charged particle picking up a U(1) phase when hopping in a loop penetrated by a magnetic field, θ_{ijk} and $\mathbf{n}_{ijk}$ parameterize the SU(2)-phase that is picked up by the electron spin when hopping around the triangle [3]. Writing the hopping part of the original Hubbard Hamiltonian

$$H_t = -\sum_{\langle ij\rangle}\mathbf{c}_i^\dagger\mathcal{A}_{ij}\mathbf{c}_j, \tag{9}$$

we can identify $\mathcal{A}_{ij}\equiv\exp(i\theta_{ij}\mathbf{n}_{ij}\cdot\boldsymbol{\sigma})$. The lowest order contribution to the polarization comes from a third order hopping process around a triangle, during which an electric spin picks up the total rotation

$$\mathcal{A}_{ij}\mathcal{A}_{jk}\mathcal{A}_{ki}\equiv\exp(-i\theta_{ijk}\mathbf{n}_{ijk}\cdot\boldsymbol{\sigma}). \tag{10}$$

This defines θ_{ijk} and $\mathbf{n}_{ijk}$. Due to translation and rotation symmetries, θ_{ijk} is the same for all bonds, and given through

$$\begin{aligned}\theta_{l,ijk} &= \cos^{-1}\left[\frac{1}{8}\left(3\cos(\theta)+5\cos(3\theta)-4\sqrt{2}\sin^2(\theta)\right)\right]\\ &= \sqrt{6}\theta + \mathcal{O}(\theta^2).\end{aligned} \tag{11}$$

The sign is ambiguous, and we have chosen $\theta_{ijk}>0$ in the second equality. The vector $\mathbf{n}_{ijk}$ depends on the bond we consider. For the bond connecting site 1 and 2 in the same unit cell (i.e., the lower edge in an upright triangle), we have

$$\mathbf{n}_{l,123}\propto\left(\sin^2(\theta)(1-2\sqrt{2}\cot(\theta)), \frac{(2\sqrt{2}\cot(\theta)-1)\sin^2(\theta)}{\sqrt{3}}, \frac{5+7\cos(2\theta)+\sin(2\theta)/\sqrt{2})}{\sqrt{6}}\right)\sim\hat{\mathbf{z}}+\mathcal{O}(\theta). \tag{12}$$

The vectors $\mathbf{n}_{231}$, $\mathbf{n}_{312}$ can be obtained from $\mathbf{n}_{123}$ through rotation by $2\pi/3$ and $4\pi/3$ around z.

Terms such as $\mathbf{S}_i\cdot\mathbf{S}_j$, $S_i^zS_j^z$, $\mathbf{S}_i\times\mathbf{S}_j$ cannot change the angular momentum along z and thus do not lead to anomalous terms. In the second order (in θ) contribution to the polarization [*cf.* Eq. (7)], we have two promising terms. The second, however, yields

$$2\theta_{ij}\theta_{ijk}(\mathbf{S}_i\cdot\mathbf{n}_{ij})(\mathbf{S}_j\cdot\mathbf{n}_{ijk}) = 2\theta_{ij}\theta_{ijk}(\mathbf{S}_i\cdot\mathbf{n}_{ij})S_j^z + \mathcal{O}(\theta^3), \tag{13}$$

and thus does not contribute to second order. The remaining term is

$$\mathbf{P}_{ij}^{(2)} = \mathbf{p}_{0,ij}2\theta^2\mathbf{S}_i\cdot\left(\mathbf{n}_{ij}-\hat{\mathbf{z}}n_{ij}^z\right)\mathbf{S}_j\cdot\left(\mathbf{n}_{ij}-\hat{\mathbf{z}}n_{ij}^z\right)+\cdots, \tag{14}$$

where we have subtracted the component of the vector $\mathbf{n}_{ij}$ along z, because it does not lead to anomalous terms.

SUPPLEMENTARY NONE 3: AMPLIFICATION HAMILTONIAN

Independent of whether we justify the existence of an anomalous term via symmetry considerations (see Microscopic model) or the microscopic derivation (Supplementary Note 2), the amplification Hamiltonian takes same form, due to symmetry constraints. Note that due to symmetry, $\mathbf{P}_{ij}$ is constrained to lie in the plane perpendicular to the bond. As the z component is irrelevant, the resulting amplification Hamiltonian depends only on the modulus of the in-plane component of the polarization $\mathbf{P}$. Taking only the relevant term, the amplification Hamiltonian is written as (note the slightly odd convention where by addition of the indices, such as $m+n$ or $1+2$, we mean that we take the vectors to those sites and add them)

$$H_{\text{amp}} = -2\theta^2\sum_{\langle mn\rangle}\mathbf{E}_{\frac{m+n}{2}}\cdot\mathbf{p}_{0,mn}(\mathbf{S}_m\cdot\mathbf{Q}_{mn})(\mathbf{S}_n\cdot\mathbf{Q}_{mn}), \tag{15}$$

where $\mathbf{Q}_{mn}=\mathbf{n}_{mn}-\hat{\mathbf{z}}n_{mn}$ is perpendicular to the bond and points outside for upright triangles and inside for upside-down triangles. In fact, it is irrelevant whether $\mathbf{Q}_{mn}$ points in or out, since $\mathbf{Q}_{mn}\to-\mathbf{Q}_{mn}$ leaves Eq. (15) unchanged. In the spin-wave picture, we have $(\mathbf{S}_m\cdot\mathbf{Q}_{mn})(\mathbf{S}_n\cdot\mathbf{Q}_{mn}) = S_m^-S_n^-(Q_{mn}^+)^2/4+\text{H.c.}+\cdots$, where $Q^\pm\equiv Q^x\pm iQ^y$. Since $\mathbf{Q}_{\text{mn}}$ points out of the upward facing triangles (they are parallel to $\mathbf{n}_{mn}$), we have $Q_{12}^+=e^{i\pi/6}/\sqrt{3}$, $Q_{23}^+=e^{5i\pi/6}/\sqrt{3}$, and $Q_{31}^+=-i/\sqrt{3}$. We end up with

$$\begin{aligned}
H_{\text{amp}} &= -\frac{1}{4}\sum_{\langle mn\rangle}\mathbf{E}_{(m+n)/2}\cdot\mathbf{p}_{0,mn}\left[a_ma_n(Q_{mn}^+)^2+\text{H.c.}\right]\\
&= -\frac{1}{4}\sum_l\Bigg[\mathbf{p}_{0,12}a_{1,l}\left(\mathbf{E}_{\frac{1+2}{2},l}a_{2,l}-\mathbf{E}_{\frac{1+2}{2},l-\frac{3}{2}}a_{2,l-3}\right)(Q_{12}^+)^2\mathbf{p}_{0,23}a_{2,l}\left(\mathbf{E}_{\frac{2+3}{2},l}a_{3,l}-\mathbf{E}_{\frac{2+3}{2},l+\frac{3}{2}}a_{3,l+2}\right)(Q_{23}^+)^2\\
&\quad+\mathbf{p}_{0,31}a_{3,l}\left(\mathbf{E}_{\frac{3+1}{2},l}a_{1,l}-\mathbf{E}_{\frac{3+1}{2},l-\frac{1}{2}}a_{1,l-1}\right)(Q_{31}^+)^2+\text{H.c.}\Bigg]\\
&= -\sum_{k,l_y}\frac{\mathbf{E}_0}{12}e^{i\Omega_0t}\Big[\mathbf{p}_{0,31}e^{i\pi/3}a_{3,k,l_y}a_{1,-k,l_y}2i\sin\left(-k\delta_1/2\right)+\mathbf{p}_{0,23}e^{-i\pi/3}a_{2,k,l_y}\left(a_{3,-k,l_y}e^{ik\delta_2/2}-a_{3,-k,l_y+1}e^{-ik\delta_2/2}\right)\\
&\quad-\mathbf{p}_{0,12}a_{1,k,l_y}\left(a_{2,-k,l_y}e^{-ik\delta_3/2}-a_{2,-k,l_y-1}e^{ik\delta_3/2}\right)\Big]+\text{H.c.},
\end{aligned} \tag{16}$$

where we have used $(Q_{12}^+)^2=\frac{1}{3}e^{i\pi/3}$, $(Q_{23}^+)^2=\frac{1}{3}e^{-i\pi/3}$, $(Q_{31}^+)^2=-\frac{1}{3}$. The minus sign between the two terms in the round and square brackets above stems from the fact that the induced polarization switches sign going from a bond to an adjacent one. Ω_0 is the frequency of the incoming radiation, $\mathbf{E}_0=\hat{\mathbf{e}}E_0$ its polarization and amplitude. If the radiation is polarized along z, at least to this order in perturbation theory, it has no effect on the TMI, thus we choose it to lay in the plane.

Recall $\mathbf{p}_{0,ij}=8ea\frac{t_{ij}t_{jk}t_{ki}}{U^3}(2\boldsymbol{\rho}_k-\boldsymbol{\rho}_i-\boldsymbol{\rho}_j)$ (where $i,j,k\in\{1,2,3\}$ and are all distinct). Then $2\boldsymbol{\rho}_1-\boldsymbol{\rho}_2-\boldsymbol{\rho}_3=(3,-\sqrt{3})/4$, $2\boldsymbol{\rho}_2-\boldsymbol{\rho}_1-\boldsymbol{\rho}_3=(0,\sqrt{3})/2$ and $2\boldsymbol{\rho}_3-\boldsymbol{\rho}_1-\boldsymbol{\rho}_2=-(3,\sqrt{3})/4$. We further define

$$\mathcal{E}\equiv\frac{\sqrt{3}eat^3}{4U^3}|E_0|, \tag{17}$$

proportional to the strength of the electric field, and go into the rotating frame with respect to the Hamiltonian $H_{\text{rot}}=\frac{\Omega_0}{2}\sum_{\alpha,l_y,p_x}a^\dagger_{\alpha,p_x,l_y}a_{\alpha,p_x,l_y}$. We arrive at

$$\begin{aligned}
H_{\text{amp}} = -\mathcal{E}\sum_{k,l_y}\hat{\mathbf{e}}\cdot\Bigg\{&\hat{\mathbf{y}}e^{i\pi/3}a_{3,k,l_y}a_{1,-k,l_y}2i\sin\left(-\delta_1k/2\right)+\frac{\sqrt{3}\hat{\mathbf{x}}-\hat{\mathbf{y}}}{2}e^{-i\pi/3}a_{2,k,l_y}\left(a_{3,-k,l_y}e^{ik\delta_2/2}-a_{3,-k,l_y+1}e^{-ik\delta_2/2}\right)\\
&+\frac{\sqrt{3}\hat{\mathbf{x}}+\hat{\mathbf{y}}}{2}a_{1,k,l_y}\left(a_{2,-k,l_y}e^{-ik\delta_3/2}-a_{2,-k,l_y-1}e^{ik\delta_3/2}\right)\Bigg\}+\text{H.c.},
\end{aligned} \tag{18}$$

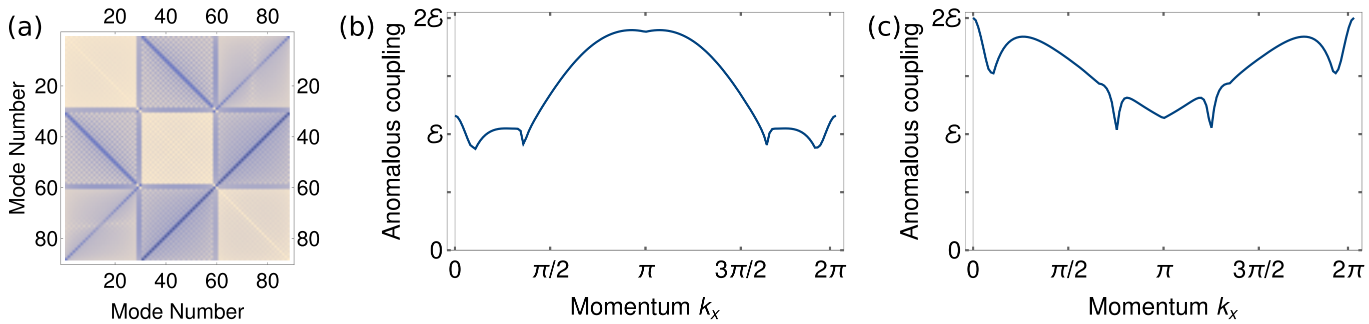

Supplementary Figure 1. **Light-magnon matrix element.** (a) Modulus of the anomalous coupling between a given pair of modes at $k = \pi$. Dark blue corresponds to a maximum of $0.038J \approx 2\mathcal{E}$, white to 0. (b) The maximum entry of the coupling matrix for wavevectors ranging from 0 to 2π. A clear maximum arises around $k \approx \pi$. (c) The same plot, repeated for a polarization along x. In this case the anomalous coupling is suppressed for modes around $k = \pi$. Parameters are the same as Fig. 1 in the main text (except for the polarization in (c)).

From this form it is clear that the terms at $\pm k$ couple, so that it is we should combine negative and positive momenta. Finally, choosing $\hat{\mathbf{e}} = \hat{\mathbf{y}}$, this leads to

$$H_{\text{amp}} = -\mathcal{E} \sum_{k>0,l_y} \left\{ \mathbf{a}^T_{k,l_y} \begin{pmatrix} 0 & \frac{1}{2}e^{-\frac{ik\delta_3}{2}} & e^{\frac{i\pi}{3}} 2i\sin(\frac{k\delta_1}{2}) \\ \frac{1}{2}e^{\frac{ik\delta_3}{2}} & 0 & -\frac{1}{2}e^{-\frac{i\pi}{3}+\frac{ik\delta_2}{2}} \\ e^{\frac{i\pi}{3}} 2i\sin\left(-\frac{k\delta_1}{2}\right) & -\frac{1}{2}e^{-\frac{i\pi}{3}-\frac{ik\delta_2}{2}} & 0 \end{pmatrix} \mathbf{a}_{-k,l_y} \right.$$
$$\left. + \frac{1}{2}\left(e^{\frac{ik\delta_2}{2}-\frac{i\pi}{3}} a_{3,k,l_y+1} a_{2,-k,l_y} + e^{-\frac{ik\delta_2}{2}-\frac{i\pi}{3}} a_{2,k,l_y} a_{3,-k,l_y+1} - e^{-\frac{ik\delta_3}{2}} a_{2,k,l_y-1} a_{1,-k,l_y} - e^{\frac{ik\delta_3}{2}} a_{1,k,l_y} a_{2,-k,l_y-1} \right) \right\} + \text{H.c.} \tag{19}$$

SUPPLEMENTARY NOTE 4: AMPLIFICATION MATRIX ELEMENT

In the section above we have derived the amplification Hamiltonian

$$H_{\text{amp}} = -\sum_{k,s,s'} \mathbf{E}_0 \cdot \mathbf{Q}_{ss'}(k) a_{k,s} a_{-k,s'} + \text{H.c.} \tag{20}$$

where here the generic indices s, s' contain both the site label α and the unit cell label l_y.

Diagonalizing the bilinear undriven Hamiltonian $H_0 = \sum_k \mathbf{a}^\dagger_k \mu_k \mathbf{a}_k = \sum_k \mathbf{b}^\dagger_k \omega_k \mathbf{b}_k$, where $\mathbf{b}_k = \mathsf{U}^\dagger_k \mathbf{a}_k$ is a vector containing the annihilation operators of the energy eigenmodes and ω_k is a diagonal matrix. Writing Eq. (20) in terms of energy eigenstates, we obtain

$$H_{\text{amp}} = -\sum_k \mathbf{b}_k \mathbf{E}_0 \cdot (\mathsf{U}^*_k \mathbf{Q}(k) \mathsf{U}_k) \mathbf{b}_k. \tag{21}$$

We can investigate the coupling strength between the various modes numerically, as is done in Fig. 1. The first conclusion, when considering the coupling matrix in the energy eigenbasis for wavevectors close to π is that the anomalous coupling in between the edge modes is among the largest. Comparable coupling strength is only achieved in between modes in differing bulk bands, as is seen from the diagonal lines in the off-diagonal blocks. This can be appreciated by thinking about the form of the bulk wavefunctions along y, which are approximately standing waves with 0 to $N_y - 1$ nodes. Since the matrix element between two bulk modes contains their product (with a constant applied field), summed over y, bulk modes with differing numbers of nodes approximately sum to zero. In between bands, the number of nodes within a unit cell changes, such that a full cancellation no longer occurs.

We next plot the maximum coupling strength between any of the modes as a function of wavevector. From this plot we conclude that the anomalous coupling is most efficient around $k \approx \pi$. This result can be understood to some degree by looking at the form of the amplification Hamiltonian Eq. (18). Choosing the polarization of the applied field to lie along y, the first term coupling sites 1 and 3 is dominant. In Fourier space this term has the functional form $\sin(k)$, such that it is largest around π, which roughly matches the shape in Fig. 1. This conclusion is strongly dependent on the polarization we choose for the applied field. We can plot the same quantities for a polarization along x, which turns off the coupling between sites 1 and 3. In this case the maximum coupling strength no longer lies around $k = \pi$, which is plotted in Fig. 1. Finally, this demonstrates one of the reasons why the

agreement between the chiral waveguide model and the microscopic two-dimensional model is so good, namely that the matrix element is near unity (in units of $2\mathcal{E}$).

As we emphasize in the main text, the anomalous coupling strength is only one of the factors that influence whether a mode pair would become unstable under driving. For example, all bulk mode pairs close to $k = \pi$ are far detuned in energy and thus cannot become unstable, regardless of the strength of their anomalous coupling.

[1] A. H. MacDonald, S. M. Girvin, and D. Yoshioka, t/U expansion for the Hubbard model, Physical Review B **37**, 9753 (1988).
[2] L. N. Bulaevskii, C. D. Batista, M. V. Mostovoy, and D. I. Khomskii, Electronic orbital currents and polarization in Mott insulators, Physical Review B **78**, 024402 (2008).
[3] S. Zhu, Y.-Q. Li, and C. D. Batista, Spin-orbit coupling and electronic charge effects in Mott insulators, Physical Review B **90**, 195107 (2014).
[4] M. Elhajal, B. Canals, R. Sunyer, and C. Lacroix, Ordering in the pyrochlore antiferromagnet due to Dzyaloshinsky-Moriya interactions, Physical Review B **71**, 094420 (2005).
